# Centering equity and justice in tobacco control

**DOI:** 10.1136/tc-2024-058767

**Published:** 2024-11-10

**Authors:** Marita Hefler

**Affiliations:** 1 Menzies School of Health Research, Charles Darwin University, Casuarina, Northern Territory, Australia; 2 School of Public Health, University of Queensland, Brisbane, Queensland, Australia

**Keywords:** Addiction, Advertising and Promotion, Advocacy

 Integrating equity and justice into tobacco control policy-making is essential, given socioeconomic, racial and other disparities in tobacco use prevalence, and the tobacco industry’s targeting of specific groups. This issue brings together papers which explore diverse issues including policy, gender issues in tobacco cultivation, retail tobacco availability, and experiences of minority groups disproportionately impacted by the tobacco epidemic, with an equity and justice lens.

Mills *et al* highlight that an equity focus often goes no further than describing inequities in tobacco use.^1^ They analysed major US tobacco control reports to provide practical and detailed recommendations to target and eliminate tobacco use inequities. The problems they highlight are not unique to the USA; while the strategies may differ, their recommendations have broad applicability for policy-makers, funding agencies, researchers and civil society. Staying in the USA, Majmudar *et al* outline a framework for partnerships between researchers and advocates to create an evidence-based approach to tobacco control policy advocacy within a large non-profit organisation.^2^ They describe how the framework was applied to inform the US Food and Drug Administration’s decision to regulate menthol-flavoured cigarettes, showing the process of identifying evidence gaps, generating evidence and outreach and engagement with policy-makers and the public. From Europe, another advocacy case study by Tsagkaris *et al* outlines lessons learned from the Youth Committee of the 2023 European Conference on Tobacco or Health including a framework for ongoing involvement in a plan to achieve a tobacco-free generation by 2040.^3^

Focusing on research practices, Maddox *et al* outline how research—and non-Indigenous researchers—can support structures and practices that perpetuate racism and inequities in tobacco control.^4^ They call for precision in language to differentiate commercial tobacco from ceremonial tobacco sacred to some Indigenous peoples, and outline guiding principles and practices journals can adopt to support Indigenous researchers and ensure research involving Indigenous peoples is ethical. In an accompanying commentary,^5^ Reid further describes how research was an integral part of colonisation, which is itself marked by genocide, ethnocide, ecocide and epistemicide (terms explained in the commentary). Reid notes the invitation for *Tobacco Control* to reflect on how we might be doing harm, and if so how we rectify the situation, noting that our response must not be decorative or performative but a genuine challenge to the status quo. *Tobacco Control* welcomes this invitation, and is developing a detailed guide for research involving or affecting Indigenous peoples, which will be integrated into our publishing processes.

The need to apply a gender lens to tobacco control issues is well recognised. Clark *et al* explore gender and family dynamics in tobacco farming, and highlight the importance of understanding these to promote better implementation of WHO Framework Convention on Tobacco Control Article 17.^6^ Moving along the supply continuum to retail, Widiantari *et al* explored e-cigarette availability to under-age youth in Bali, Indonesia. They found around a quarter of vape stores were within 250 m of a school, and nearly half of vape store owners sold to minors.^7^ Also in Indonesia, Amalia *et al* describe a disturbing example of a tobacco company using cutouts to undermine graphic health warning labels.^8^ In a completely different context, Pätsi *et al* examined socioeconomic differences in retailer density in Finland, which is a tobacco control leader and has a tobacco retailer licence system.^9^ Despite having relatively low-income inequality, tobacco retailer density was higher in lower sociodemographic areas, highlighting the opportunity to reduce inequities through changes to the retailer licensing system.

A UK study explores the impact of vaping on socioeconomic inequalities in smoking cessation and relapse. Using longitudinal data from 2016 to 2020, Hardie *et al*^10^ found that e-cigarette use may be particularly useful to support people who smoke and experience disadvantage to stop. However, they conclude that other supports or aids may be needed to reach those who experience higher levels of disadvantage. Also in the UK, which bans point-of-sale displays for tobacco but not e-cigarettes, Parnham *et al*^11^ show the need for consistent policies across different products. They found that among youth aged 11–18, noticing e-cigarettes increased from 2018 to 2022, and young people were becoming more likely to purchase e-cigarettes from small shops. Moving to the older end of the age spectrum, Rubenstein *et al* examined USA Population Assessment of Tobacco and Health (PATH) study data to understand perceptions of e-cigarettes among people aged 55 or older compared with younger adults.^12^ Older adults who smoke were less likely to perceive cigarettes as harmful or very harmful, and more likely to rate e-cigarettes as harmful or very harmful—highlighting the need for accurate communication among this age group.

Also in the USA, Budenz *et al* estimated the association between subgroups of sexual and gender minority (SGM) young adults, race/ethnicity, SGM-related discrimination, SGM identity connection and tobacco use.^13^ Their study shows the importance of intersectional issues for this population with high tobacco use prevalence, with higher levels of discrimination associated with multiple tobacco use. Conversely, identity connectedness was found to possibly be protective against tobacco use. Also on the topic of minority disparities, Burciaga Valdez and Encinosa examined racial and ethnic disparities in medical expenditure attributable to smoking in the USA. They found that although White adults had higher ever-smoked rates and minorities made more smoking cessation attempts, smoking-related medical spending for minorities was twice as high.^14^ One issue common across many groups who use tobacco products is experiencing financial hardship. In the USA, despite the increased financial pressures many experienced during COVID-19, commercial tobacco sales increased. Zarei *et al* used a nationally representative online sample to collect data about participants’ experiences of hardship and receiving discount coupons for tobacco products.^15^ Showing the tobacco industry’s resourcefulness at exploiting hardship, they found that more than 20% of participants who used tobacco products and had previously used discount coupons received more coupons during the pandemic. The utility of tobacco coupons in facilitating established smoking among young adults aged 18-24 is demonstrated by Siegel *et al*, using five waves of US PATH data from 2013-2019.^16^

Three papers explore priority groups and equity in the tobacco endgame. Puljevic *et al* summarise the research on likely impacts and perceptions of tobacco endgame policies among eight priority population groups.^17^ They found that the literature is dominated by studies of very low-nicotine cigarettes and more evidence is needed to understand acceptability and likely effectiveness of policies among these groups. Trigg *et al* go some way to addressing that gap with a qualitative exploration of acceptability of tobacco retailer reduction, very low nicotine cigarettes and subsidised vaping support among residential alcohol and other drug treatment clients.^18^ They found the most accepted policy was subsidised nicotine vaping products. Ait Ouakrim *et al* simulated the impact of Aotearoa New Zealand’s (now repealed) tobacco action plan.^19^ Their modelling found that a tobacco endgame strategy would likely have a major impact on both overall health status and reducing inequities between Māori and non-Māori people. Their study shows that perhaps the greatest equity and justice approach is an effective, multipronged, well-enforced tobacco endgame strategy.

Finally, three studies put the spotlight for tobacco-caused harms on the industry. Sy estimates the annual global cost of ecosystem losses and waste management of plastic waste from filtered commercial cigarettes at US$26 billion.^20^ Tabbakh *et al* report how messages about climate, pollution and social justice harms were found to be smoking cessation motivators among a majority of participants in a sample of Australian adults who smoke.^21^ While this appears to be an untapped opportunity, Sy *et al* remind us that the tobacco industry is one of the world’s most profitable while it largely escapes bearing the costs of the harm it causes to people and the environment. They outline several mechanisms by which the tobacco industry could be held fully liable for these costs.^22^ Enacting these would be an important step towards achieving justice, given that the devastation wrought by the industry is largely borne by both countries and people of lower income, as profits accrue to the wealthy.

## References

[1] Mills SD, Rosario C, Yerger VB (2024). Recommendations to advance equity in tobacco control. Tob Control.

[2] Majmundar A, Xue Z, Asare S (2024). An advocacy-research collaboration model to inform evidence-based tobacco control efforts. Tob Control.

[3] Tsagkaris C, Arranz B, Yared W (2024). How can youth involvement in public health conferences promote a tobacco-free generation? Lessons from the ECToH 2023’s Youth Committee. Tob Control.

[4] Maddox R, Drummond A, Kennedy M (2024). Ethical publishing in ‘indigenous’ contexts. Tob Control.

[5] Reid P (2024). Valuing indigenous wisdom: invited comment. Tob Control.

[6] Clark M, Cunguara B, Bialous S (2024). Foregrounding women and household dynamics to inform article 17: a qualitative description analysis of tobacco farming households in mozambique. Tob Control.

[7] Widiantari NK, Kurniasari NMD, Trapika I (2024). Vape store density and proximity to schools in denpasar. Tob Control.

[8] Amalia B, Nguyen N, Welding K (2024). Another day, another tobacco company’s devious behaviour: cutout health warning labels on Indonesian cigarette packs. Tob Control.

[9] Pätsi S-M, Toikka A, Ollila H (2024). Area-level sociodemographic differences in tobacco availability examined with nationwide tobacco product retail licence data in Finland. Tob Control.

[10] Hardie I, Green MJ (2024). Vaping and socioeconomic inequalities in smoking cessation and relapse: a longitudinal analysis of the UK household longitudinal study. Tob Control.

[11] Parnham JC, Vrinten C, Cheeseman H (2024). Changing awareness and sources of tobacco and E-cigarettes among children and adolescents in great Britain. Tob Control.

[12] Rubenstein D, Denlinger-Apte RL, Cornacchione Ross J (2024). Older age is associated with greater misperception of the relative health risk of E-cigarettes and cigarettes among US adults who smoke. Tob Control.

[13] Budenz A, Gaber J, Crankshaw E (2024). Discrimination, identity connectedness and tobacco use in a sample of sexual and gender minority young adults. Tob Control.

[14] Burciaga Valdez R, Encinosa W (2024). Racial and ethnic disparities in healthcare costs and outcomes of cigarette smoking in USA: 2008–2019. Tob Control.

[15] Zarei K, Hamilton-Moseley KR, Chen-Sankey J (2024). Financial hardship during the COVID-19 pandemic and increased receipt of commercial tobacco discount coupons among US adults who use commercial tobacco tobacco control in this issue. Tob Control.

[16] Siegel LN, Cook S, Oh H (2024). The longitudinal association between coupon receipt and established cigarette smoking initiation among young adults in USA. Tob Control.

[17] Puljević C, Feulner L, Hobbs M (2024). Tobacco endgame and priority populations: a scoping review. Tob Control.

[18] Trigg J, Rich J, Williams E (2024). Perspectives on limiting tobacco access and supporting access to nicotine vaping products among clients of residential drug and alcohol treatment services in Australia. Tob Control.

[19] Ait Ouakrim D, Wilson T, Waa A (2024). Tobacco endgame intervention impacts on health gains and māori:non-māori health inequity: a simulation study of the Aotearoa/New Zealand tobacco action plan. Tob Control.

[20] Sy DK (2024). Tobacco industry accountability for marine pollution: country and global estimates. Tob Control.

[21] Tabbakh T, Mitsopoulos E, Nuss T (2024). Messages about climate, pollution and social justice harms of tobacco as motivators to quit: an untapped communication opportunity?. Tob Control.

[22] Sy D, El-Awa F, Al-Lawati JA (2024). Towards health with justice: making the tobacco industry accountable through administrative liability. Tob Control.

